# Apathy and suicide-related ideation 3 months after stroke: a cross-sectional study

**DOI:** 10.1186/s12883-015-0323-3

**Published:** 2015-04-23

**Authors:** Wai Kwong Tang, Lara Caeiro, Chieh Grace Lau, Huajun Liang, Vincent Mok, Gabor S Ungvari, Ka Sing Wong

**Affiliations:** Department of Psychiatry, Shatin Hospital, The Chinese University of Hong Kong, Shatin, NT, Hong Kong, SAR China; Faculty of Medicine, Institute of Molecular Medicine, University of Lisbon, Lisbon, Portugal; Department of Medicine and Therapeutics, The Chinese University of Hong Kong, Hong Kong, SAR China; School of Psychiatry & Clinical Neurosciences, University of Western Australia, Perth, Australia; University of Notre Dame Australia / Marian Centre, Perth, Australia

**Keywords:** Apathy, Suicide-related ideation, Stroke

## Abstract

**Background:**

Both apathy and suicide are common in poststroke patients. However, the association between poststroke apathy and suicide-related ideation (SI) in Chinese stroke patients is not clear and poorly understood. The aim of this study was to examine the association between apathy and SI in stroke.

**Methods:**

A cross-sectional study was conducted to investigate the association in 518 stroke survivors from Acute Stroke Unit of the Prince of Wales Hospital in Hong Kong. Geriatric Mental State Examination-Version A (GMS) and Neuropsychiatric Inventory-apathy subscale (NPI-apathy) were employed to assess poststroke SI and apathy, respectively. Patients’ clinical characteristics were obtained with the following scales: the National Institutes of Health Stroke Scale (NIHSS), the Mini-Mental State Examination (MMSE), and the Geriatric Depression Scale (GDS).

**Results:**

Thirty-two (6.2%) stroke survivors reported SI. The SI group had a significantly higher frequency of NPI-apathy than the non-SI group (31.2% vs 5.3%, p < 0.001). The SI group also had higher GDS scores (10.47 ± 3.17 vs 4.24 ± 3.71, p < 0.001). Regression analysis revealed that NPI-apathy (OR 2.955, 95% CI 1.142-7.647, p = 0.025) was a significant predictor of SI. The GDS score also predicted SI (OR 1.436, 95% CI 1.284-1.606, p < 0.001).

**Conclusions:**

The current findings show that poststroke apathy is an independent predictor of SI 3 months after stroke. Early screening for and intervention targeting apathy through medication and psychological treatments may be necessary to improve stroke patients’ apathy and reduce SI.

## Background

Suicide-related ideation (SI) is any self-reported thoughts of engaging in suicide-related behavior [1]. SI affects 7-15% of stroke patients [2-4]. The annual incidence of suicide in stroke is 83/100,000[5]. Poststroke suicide (PSSI) is correlated with impaired cognitive and sensory functions [3], sleep disturbances [6], depression [2,7] and recurrent stroke [3,4].

Apathy is a common neuropsychiatric syndrome following stroke (poststroke apathy) [8-10]. It is defined as a combination of lack of emotion, interest, concern and motivation [11] manifesting in poor engagement with significant others and in social activities, and loss of pleasure and usual interests [12]. The prevalence of poststroke apathy is 38-43% [8-10]. Poststroke apathy is associated with impaired cognitive function [13-16] and physical abilities [13,15] and depressive symptoms [13,14]. It has a negative impact on recovery [8,15], particularly on daily functioning [15,17].

Apathy predicts suicide [18,19] in other disease affecting the central nervous system. In one study, patients with spinal cord injuries who committed suicide were significantly more apathetic than their non-suicidal counterparts matched according to age and sex, and apathy was predictive of suicide [18]. Suicide attempts in Huntington’s disease are related to symptoms of apathy [19]. To the best of our knowledge, the relationship between poststroke apathy and SI has not been reported. Thus, the aim of this study was to examine the association between SI and apathy in stroke. We hypothesized that poststroke apathy would be associated with SI.

## Methods

### Patients

Two thousand one hundred and six stroke patients were admitted to the Acute Stroke Unit of the Prince of Wales Hospital between December 2006 and September 2011. This is a university-affiliated regional hospital serving a population of 800,000 in the Shatin district of the Hong Kong SAR, China.

The inclusion criteria for the study were (1) Chinese ethnicity; (2) Cantonese as the primary language; (3) age of 18 years or above; and (4) well-documented (clinical presentation and CT scan of the brain) stroke occurring within seven days before the index admission. The exclusion criteria included (1) transient ischemic attack, cerebral haemorrhage, subdural hematoma, or subarachnoid haemorrhage; (2) history of any central nervous system (CNS) disease other than stroke; (3) severe cognitive impairment, as defined by a Mini-Mental State Examination (MMSE) [20] score of less than 20; (4) aphasia; (5) loss to follow-up; (6) physical frailty preventing attendance at the follow-up interview; (7) severe auditory or visual impairment; and (8) recurrent stroke before the follow-up assessment.

### Measurements

The study protocol was approved by the Clinical Research Ethics Committee of the Chinese University of Hong Kong. This institution adheres to the principles laid down in the Declaration of Helsinki. All participants signed a consent form.

Patients’ socio-demographic and clinical characteristics were obtained by a trained research assistant. The severity of stroke was assessed with the National Institutes of Health Stroke Scale (NIHSS) [21] by a research nurse on the day of admission.

SI was assessed with the Geriatric Mental State Examination–Version A [22] 3 months after the index stroke by a trained research assistant. An affirmative response to the statement “Felt suicidal or had wished to be dead sometime in the past month” indicated SI [22].

The Neuropsychiatric Inventory-apathy subscale (NPI-apathy) was designed to assess psychopathology in patients with dementia and other neuropsychiatric disorders [23]. Its Chinese version (CNPI) has been used in Alzheimer’s disease [24,25]. The CNPI, administered by a psychiatrist, is a structured interview with a caregiver who is familiar with the patient. The frequency of symptoms is rated on a scale from 1 to 4 (1 = occasionally; 2 = often; 3 = frequently; 4 = very frequently) and their severity is scored 1 to 3 (1 = mild; 2 = moderate; 3 = severe). The magnitude of apathy is calculated as the product of severity and frequency (severity of apathy multiplied by its frequency) [23]. Cronbach’s α for the overall reliability of the CNPI has been reported as 0.84, with the interclass correlation coefficient of all subscales at >0.9 [26]. Its concurrent validity is good, as shown by an acceptable correlation between the NPI scores and the Chinese version of the Alzheimer’s disease behavioral pathology rating scale and the Chinese Hamilton rating scale of depression [26].

Global cognitive functions were evaluated by a trained research assistant with the Chinese version of the MMSE [20]. Cronbach’s α and the test-retest reliability of the Chinese version of the MMSE are 0.86 and 0.78, respectively; its inter-rater reliability, as measured by intra class correlation, is 0.99 [20].

The Chinese version of the 15-item Geriatric Depression Scale (GDS) [27] was used by a trained research assistant to rate depressive symptoms. The GDS has been used to evaluate depressive symptoms [28,29] with good psychometric properties. Cronbach’s alpha and the test-retest reliability of the GDS are 0.89 and 0.85, respectively [30].

### Statistical analysis

Statistical analyses were performed using IBM SPSS Statistics, Version 20 (SPSS Inc., Chicago). Descriptive data are presented here as means or proportions as appropriate. Continuous variables were analysed with independent t-tests for comparing SI and non-SI groups; chi square tests were used for categorical variables. Univariate analysis was used to determine the difference between SI and non-SI groups with respect to the demographic and clinical variables. Variables with p < 0.1 in the univariate analysis were entered into the logistic regression (forward mode) to examine their relationships with SI. The level of significance was set at 0.05 (two-tailed).

## Results

Five hundred and eighteen (24.6%) stroke patients met the inclusion criteria. Patients excluded from the study had higher NIHSS scores (5.01 ± 5.10 vs 3.73 ± 3.55; p < 0.001). The age (67.65 ± 12.14 vs. 66.70 ± 10.15; p = 0.108) and sex distribution (57.6 % vs. 57.3% male; p = 0.916) of the excluded and included groups was nearly identical.

Thirty-two (6.2%) of the stroke survivors reported SI. No statistical differences were found between the SI and non-SI groups in terms of sex, age, marital status, education, and MMSE score. The SI group had a significantly higher GDS score (10.47 ± 3.17 vs. 4.24 ± 3.71, p < 0.001) and frequency of NPI-apathy (31.2% vs. 5.3%, p < 0.001). The Spearman’s correlation between GDS score and NPI-Apathy score was 0.269, p < 0.001. The difference in the NIHSS score was of borderline significance (p = 0.051) (Table 1).

**Table 1:** **Patients’ demographic and clinical characteristics in suicide-related ideation (SI) and non SI groups**

**Variable**	**SI N = 32**	**Non SI N = 486**	**P value**
Age*	66.25 ± 8.86	66.73 ± 10.24	0.795
Education (years)*	5.71 ± 4.37	6.02 ± 4.85	0.730
Female	18(56.2)	203(41.8)	0.109
Married	26(83.9)	375(79.6)	0.567
NIHSS score*	4.91 ± 4.06	3.65 ± 3.50	0.051
MMSE score*	26.44 ± 3.16	27.06 ± 2.67	0.211
GDS score*	10.47 ± 3.17	4.24 ± 3.71	<0.001
NPI-apathy (%)	10(31.2)	26(5.3)	<0.001

GDS = Geriatric Depression Scale; MMSE = Mini Mental State Examination; NIHSS = National Institute of Health Stroke Scale; NPI-apathy = Neuropsychiatric Inventory-apathy subscale; SI = suicide-related ideation.

* mean ± SD, independent t-test; otherwise chi square test is used.

In the regression analysis, NIHSS, GDS, and NPI-apathy were entered into the model. GDS (OR 1.436, 95% CI 1.284-1.606, p < 0.001) and NPI-apathy (OR 2.955, 95% CI 1.142-7.647, p = 0.025) were both significant predictors of SI (Table 2).

**Table 2:** **Logistic regression, forward mode**

**Variable**	**Odd ratio**	**95% Confident interval**	**P value**	**Nagelkerke R square**
Model 1				0.348
GDS score	1.436	1.284-1.606	<0.001	
NPI-apathy (%)	2.955	1.142-7.647	0.025	

GDS = Geriatric Depression Scale; NPI-apathy = Neuropsychiatric Inventory-apathy subscale.

## Discussion

To the best of our knowledge, this is the first report of a significant association between apathy and SI in stroke. Poststroke apathy was an independent predictor of SI.

At first sight, it would appear that apathy and SI are both manifestations of an underlying depression, which is the root cause of SI. However, depression cannot be the whole explanation because the association between apathy and SI remains even after accounting for the concurrent depression. This association may also be explicable in terms of lesion location. Poststroke apathy is associated with pontine infarcts [31]. Deficits in serotonergic and noradrenergic neurotransmission have been implicated in the pathogenesis of SI. A recent positron-emission tomography study [32] indicated an association between reduced serotonin transporter binding in the midbrain/pons area and suicide attempts in depression. In addition, suicidal depressive patients were found to have noradrenergic dysregulation in the locus coeruleus [33]. Further, higher volume of frontal white matter hyperintensities is associated with apathy in both Alzheimer’s disease [34] and suicidal patients with a variety of major affective disorders, such as bipolar disorder and unipolar major depressive disorder [35]. White matter hyperintensity is a predictor of suicide attempts in depression [36] and bipolar disorder [36]. Hence, it is possible that underlying pontine lesions or white matter hyperintensities and the subsequent neurochemical dysfunction contributes to the development of both apathy and SI. Recently, a study pointed out that dysthymic temperament together with deep white matter hyperintensity is associated with suicidal risk [37]. If this finding could be replicated in stroke survivors, it will help to discriminate those with high suicide risk from others.

In view of the association between PSSI and poststroke apathy, apathy should be regularly screened and vigorously treated in stroke patients [38]. Validated screening instruments for apathy, such as the WHO-5 [39] and the Apathy Evaluation Scale, clinician version [24], could be useful for this purpose. Nefiracetam in doses of 900 mg/day for 4 weeks was related to a significant reduction in apathy symptoms in poststroke depression [40]. Toyoda et al. [41] showed that Cilostazol administered for 6 months was also effective in poststroke apathy. Moreover, in a recent study, escitalopram or problem solving therapy has been shown to be effective in the prevention of poststroke apathy [42]. Psychological interventions, such as motivational interviewing [43], milieu therapy [44] and occupational therapy [45] have reduced apathy in traumatic brain injury [43] and dementia [44,45]. Longitudinal research on the course and pharmacological and psychosocial treatment of poststroke apathy is clearly warranted.

The frequency of SI (6.2%) found in this study was slightly lower than that reported earlier [4]. The percentage of suicidal idea was 9.8% at 3-month and 14% at 15-month after stroke in the aforementioned study. The lower frequency in this study could be explained by the different instruments used (single item of Beck depression questionnaire in this study) [4]; Frequency of apathy was 6.9% in this study sample, which is close to the figure in our previous study [31]. The prevalence of poststroke apathy ranges from 19 to 55% [14,15]. This large variability may be due to differences in the selection of patients, diagnostic criteria or the time elapsed since the stroke [15]. The previous study diagnosed apathy with the relevant item of the Beck Depression Inventory, whereas the current study used the CNPI. In this study, stroke survivors with SI presented with more depressive symptoms, which is consistent with findings in the literature [2,3,46].

### Limitations of the study

This study had two main limitations. First, because of the cross-sectional design, the causality of the relationship between poststroke apathy and PSSI could not be explored. Second, the exclusion of physically frail, aphasic, and perceptually impaired stroke survivors probably reduced the generalizability of the findings. Third, the suicidal problems in these stroke patients were not assessed using specific instruments such as Beck Hopelessness Scale [47]. Fourth, the sample size of group with both SI and apathy in this study is small. Fifth, imaging data were not available.

## Conclusion

Apathy is an independent predictor of suicide-related ideation 3 months after stroke. Future research should pay effort on whether early screening for and treatment of apathy with pharmacological agents and psychological interventions have been found to improve apathy and may reduce suicide-related ideation.

## Abbreviations

CNPI: Chinese version
GDS: the Geriatric Depression Scale
GMS: Geriatric Mental State Examination-Version A
MMSE: Mini-Mental State Examination
NIHSS: Institutes of Health Stroke Scale
NPI: Neuropsychiatric Inventory
PSSI: Poststroke suicide
SI: Suicide-related ideation
